# Effects of Aluminum Sulfate and Quicklime/Fluorgypsum Ratio on the Properties of Calcium Sulfoaluminate (CSA) Cement-Based Double Liquid Grouting Materials

**DOI:** 10.3390/ma12081222

**Published:** 2019-04-14

**Authors:** Yuli Wang, Jie Yu, Junjie Wang, Xuemao Guan

**Affiliations:** 1 School of Materials Science and Engineering, Henan Polytechnic University, Jiaozuo 454003, China; wangyuli@hpu.edu.cn (Y.W.); yu1994jie@163.com (J.Y.); guanxuemao@hpu.edu.cn (X.G.); 2 Division of Engineering, New York University Abu Dhabi, Abu Dhabi, P.O. Box 129188, UAE

**Keywords:** double liquid grouting material, calcium sulfoaluminate (CSA) cement, quicklime, aluminum sulfate, fluorgypsum, hydration process

## Abstract

Grouting materials are used frequently in grouting reinforcement projects, such as mining and coastal engineering. Double liquid grouting materials are mostly used because of the fast setting and high early strength properties when the two slurries are mixed together but high fluidity when the two slurries are separated. In our study, double liquid grouting materials were developed from CSA cement (slurry A), quicklime and fluorgypsum (slurry B). Aluminum sulfate was added in slurry B in order to counteract any adverse effects caused by the fluorgypsum, such as the decreased early compressive strength and the prolonged setting time. The effects of aluminum sulfate content and the quicklime/fluorgypsum ratio on the setting time, hydration heat, and compressive strength of the double liquid grouting materials were investigated, and the hydration products were characterized through thermogravimetry-differential thermal analysis (TG-DTA), X-ray Diffraction (XRD), and Scanning Electron Microscope (SEM) tests. The results show that the addition of aluminum sulfate can shorten the setting time and increase compressive strength at both early and later ages. Considering the setting time and compressive strength of double liquid grouting material at the same time, the optimum content of aluminum sulfate was found to be 2%, and the optimum ratio of quicklime/fluorgypsum was found to be 2:8. The values of the optimum content of aluminum sulfate and ratio of quicklime/fluorgypsum were verified from theoretical analysis.

## 1. Introduction

Grouting materials can be used to strengthen and consolidate the loose rocks around mining wells or strengthen the marine bed in coastal engineering [1]. The requirements of grouting materials include high liquidity, fast setting and high early strength. In most cases, a single liquid grouting material does not meet all these requirements, this is because a single liquid grouting material with a high liquidity might not have a fast setting ability, and there could also be leakage from the cracks in the rocks in this case [2,3,4]. So, double liquid grouting materials were developed. Double liquid grouting materials are composed of two separate slurries (usually named as slurry A and slurry B) [4,5], which have a very high liquidity separately but harden quickly when they are mixed together. The two slurries are injected separately into the rocks and they meet each other and harden quickly at the target location.

Grouting materials were first used by Charles Bering in 1802 and this method has since been developed for two decades and become an important application method in underground projects and mining projects for enhancing strength and preventing leakage [5]. Calcium sulfoaluminate (CSA) cement is the most selected cement for grouting materials compared to Portland cement because CSA cement has faster setting ability, higher early strength and some volume expansion after hardening [6,7,8]. With the increase of water/cement ratio as required by high liquidity, the strength of CSA cement-based grouting material decreases and the setting time increases. This might not meet the requirements for grouting materials in some projects. In this case, some cement additives are needed [9,10,11,12]. For example, lithium carbonate can increase the hydration products and modify the microstructure of CSA cement-based grouting material [13,14]. Besides, many nano-particles like nano-CuO, nano-SiO_2_, nano-TiO_2_, nano-Al_2_O_3_ and nano-carbon tubes were used to modify and accelerate the hydration process of CSA cement-based grouting materials [15,16,17,18,19]. Ettringite could act as a role in destroying the structure in the cementitious system [20,21], but a recent study has shown that, for sulphoaluminate cement-based grouting materials, the addition of 4% superfine ettringite can increase the 4-hour compressive strength by 380% and shorten initial setting time by 55.6% [22].

Fluorgypsum is a by-product of making hydrofluoric acid (HF), as in Equation (1), and 3.6 tons of fluorgypsum was produced for every ton of HF. Fluorgypsum has some shortages when it is used as cementitious materials, including slower hydration rate and lower early age strength [23,24], compared to nature gypsum although their hydration equation with water are the same as in Equation (2). This indicates the fluorgypsum could be used in the slurry B [4] to replace the natural gypsum. Slurry B is usually composed of gypsum, water and quick lime [4]. Slurry A is usually CSA cement and water [4]. The hydration of the main minerals in CSA clinker is as in Equations (3) and (4), and the ettringite (3CaO∙Al_2_O_3_∙3CaSO_4_∙32H_2_O) and aluminum gel (Al_2_O_3_∙3H_2_O) as in Equation (3) are formed when there is enough gypsum [25,26]. When there is not enough gypsum, the reaction as in Equation (5) could happen and the AFm (3CaO∙Al_2_O_3_∙CaSO_4_∙12H_2_O) could be formed. Given the formation of aluminum gel, when CaO and gypsum were added, they will react with the aluminum gel and form additional ettringite as shown in Equation (6). In order to reduce the use of nature resources [27,28,29,30,31,32,33,34] and increase the use of recycled materials [35,36,37], fluorgypsum was used in this study. Besides, adding slaked lime and aluminum sulfate can promote the formation of ettringite and C-S-H in masonry mortar [38]. Theoretically, as in Equation (7), the addition of aluminum sulfate to the slurry B can form ettringite, and the ettringite can affect the double liquid grouting material [22]. The influence of aluminum sulfate amount and the quicklime/fluorgypsum ratio in slurry B on the properties of CSA cement based double liquid grouting materials was investigated through a set of tests including setting time, hydration heat, compressive strength, thermogravimetry-differential thermal analysis (TG-DTA), X-ray diffraction (XRD), and scanning electron microscope (SEM) tests. The theoretical analysis of the optimum content of the ratio of quicklime/fluorgypsum and aluminum sulfate amount were carried out.

H_2_SO_4_ + CaF_2_ → 2HF + CaSO_4_ (1)

CaSO_4_ + 2H_2_O → CaSO_4_∙2H_2_O (2)

3CaO∙3Al_2_O_3_∙CaSO_4_ + 2(CaSO_4_∙2H_2_O) + 36H_2_O → 3CaO∙Al_2_O_3_∙3CaSO_4_∙32H_2_O + 2(Al_2_O_3_∙3H_2_O) (3)

2CaO∙SiO_2_ + nH_2_O → C-S-H + Ca(OH)_2_ (4)

3CaO∙3Al_2_O_3_∙CaSO_4_ + 18H_2_O → 3CaO∙Al_2_O_3_∙CaSO_4_∙12H_2_O + 2(Al_2_O_3_∙3H_2_O) (5)

3CaO + Al_2_O_3_∙3H_2_O + 3(CaSO_4_∙2H_2_O) + 23H_2_O → 3CaO∙Al_2_O_3_∙3CaSO_4_∙32H_2_O (6)

Al_2_(SO_4_)_3_∙18H_2_O + 6CaO + 14H_2_O → 3CaO∙Al_2_O_3_∙3CaSO_4_∙32H_2_O (7)

## 2. Materials and Methods 

### 2.1. Materials

The CSA clinker was the binder material in slurry A. The quicklime and fluorgypsum were the binder materials in slurry B. The content of effective CaO in quicklime was 76.5%. In slurry B, the aluminum sulfate with a chemical formula of Al_2_(SO_4_)_3_∙18H_2_O was added as an additive. The effective dosage in the chemical reagent of aluminum sulfate was 99%. The specific surface areas of CSA clinker and fluorgypsum were 350 m^2^/kg and 395 m^2^/kg. The chemical composition of raw materials was measured according to methods for chemical analysis of cement (GB/T 176-2017) [39]. The compositions of CSA clinker and fluorgypsum are shown in Table 1, Table 2 and Table 3. The main minerals in CSA clinker are 3CaO∙Al_2_O_3_∙CaSO_4_ or Ca_4_Al_6_SO_16_ and 2CaO∙SiO_2_. The mix design of double liquid grouting materials is shown in Table 4. In each mix, the two slurries were mixed together by a ratio of 1:1. Slurry A was kept same in all mixtures, and in slurry B the quicklime fluorgypsum ratio and aluminum sulfate content were changed in different mixtures to consider the influence of the quicklime/fluorgypsum ratio and content of aluminum sulfate varied in order to study their influences on properties of grouting materials. Based on the reported results in literature [4], when water/cement ratio was 1:1, 20% of the ultra-fine quicklime and 80% of ultra-fine anhydrite gave the highest compressive strength of CSA-based double liquid grouting material [4]. So, in mixtures AS0-AS4, the ratio of quicklime/fluorgypsum was kept as the same as 2:8, and the content of aluminum sulfate was increased from 0% to 4%. In mixtures QL0-QL40, the content of aluminum sulfate was kept same as 2%, and the content of quicklime increased from 0% to 40%.

### 2.2. Test Methods

The initial and final setting time of double liquid grouting materials was tested according to ASTM C191-13 with a standard Vicat apparatus [40]. Cubic samples with 70.7 mm × 70.7 mm × 70.7 mm were prepared for compressive strength test. The samples were demolded after 4 h and cured under a standard condition (20 °C, >95% R.H.) until tests. The compressive strength tests were conducted at ages of 1, 3, 7 and 28 days. One gram of the powder sample (size < 0.063 mm) was used for the XRD test each time. The TG-DTA tests were conducted with the temperature increasing from 20 °C to 800 °C at a rate of 10 °C/min in N_2_ environment, and the nitrogen flow was 10 mL/min. The XRD tests were conducted using a Rigaku SmartLab X-ray diffractometer (Rigaku, Samart-lab, Tokyo, Japan) with an operating voltage of 40 kV and a current of 150 mA. The scanning rate was 10°/min from 5° to 70°. MERLIN CompactField Emission Scanning Electron Microscope (Merlin Compact, Carl Zeiss NTS GmbH, Jena, Germany) was used for SEM observations. The samples were gold coated and the observation was conducted under high vacuum with a voltage of 15 kV and a working distance of 10 mm. The detailed test procedures of hydration heat, compressive strength, DTA-TG, XRD and SEM can also be found in our previous paper [13]. 

## 3. Results

### 3.1. Setting Time

**Figure 1:**
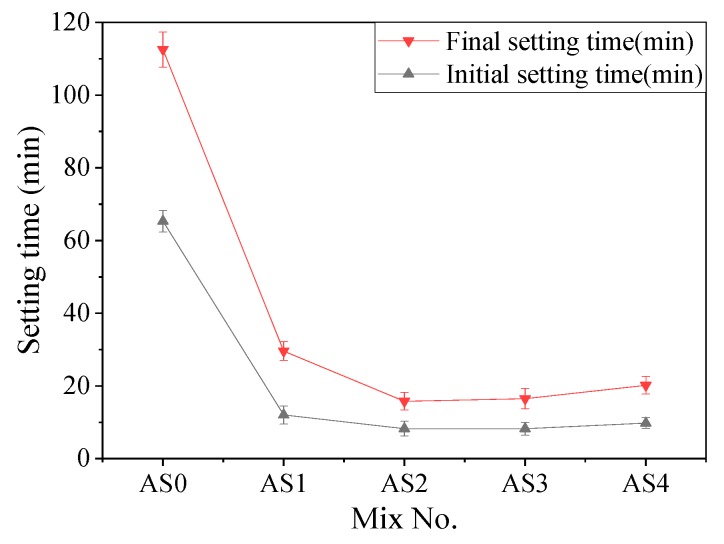
Effect of aluminum sulfate on the setting time.

**Figure 2:**
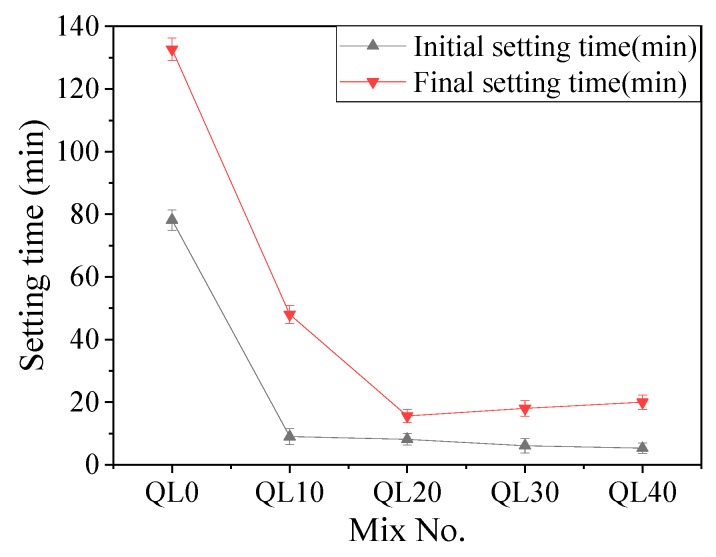
Effect of quicklime on the setting time.

Table 5 shows the initial and final setting time of the slurry A with different content of aluminum sulfate, and they were all more than 7 h. Slurry B did not set by itself. When the two slurries were mixed together, the influence of aluminum sulfate and quicklime on the setting time of the double liquid grouting materials is shown in Figure 1 and Figure 2. Figure 1 shows the effect of aluminum sulfate on the initial and final setting time of the mixtures with different contents of aluminum sulfate. Compared with the control group (AS0), the initial and final setting time of AS1 decreased significantly. The initial and final setting of AS2 were reduced from 65 and 112 min to 8 and 16 minutes compared with AS0. Further increase of aluminum sulfate did not change the setting time significantly. Figure 2 shows the effect of quicklime on the initial and final setting time of the mixtures with 0–40% of quicklime. Similar as the trend in Figure 1, Compared with QL0, the initial and final setting time of QL10 decreased significantly. The initial and final setting time of QL10 were decreased from 78 and 133 min to 8 and 16 min compared with QL0. The decreasing effect of aluminum sulfate and quicklime on setting time of double liquid grouting materials can be attributed to the formation of initial ettringite with the reaction as shown in Equation (7) [41,42]. Further increase of aluminum sulfate and quicklime might increase the formation of ettringite but the effect of more ettringite on the setting time might be limited.

### 3.2. Compressive Strength

**Figure 3:**
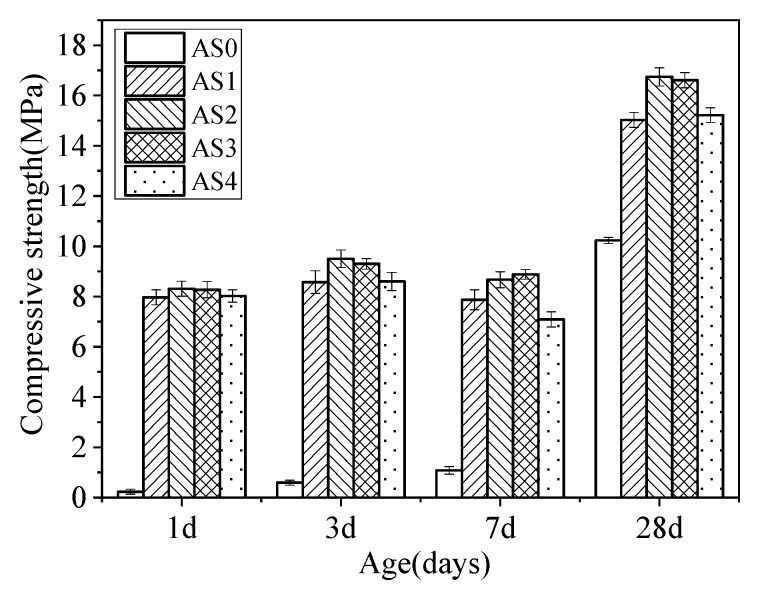
Effect of aluminum sulfate on the compressive strength at different ages.

Figure 3 shows the effect of aluminum sulfate on the compressive strength of different mixtures at ages of 1, 3, 7 and 28 days. It can be seen that the addition of aluminum sulfate is essential to get a high early compressive strength. The mixture of AS0 had a very low compressive strength at early ages up to 7 days. Because gypsum, under a standard curing condition with >95% R.H., will lose its strength gradually. Compared with AS0, the compressive strength of AS1 increased by more than 3300%, 1300%, 600% and 46% at ages of 1, 3, 7 and 28 days respectively. In all groups in which aluminum sulfate was added, the compressive strength of AS2 was almost the highest at all ages. So optimum content of aluminum sulfate in the double liquid grouting materials with a water/cement ratio of 1.0 is 2%.

**Figure 4:**
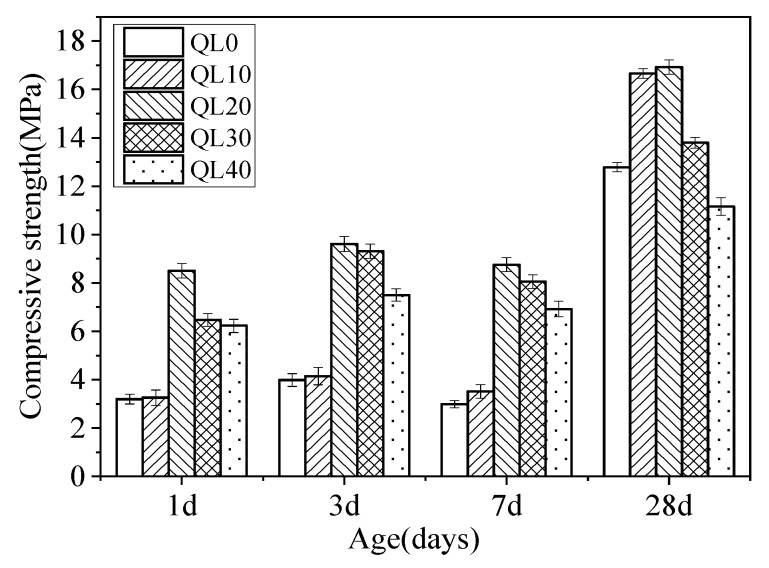
Effect of quicklime on the compressive strength at different ages.

The effects of quicklime content on the compressive strength of different mixtures at ages of 1, 3, 7 and 28 days are shown in Figure 4. Compared with QL0, the compressive strength of QL20 increased by 165%, 140%, 192% and 32% respectively at the ages of 1, 3, 7 and 28 days. For CSA cement, more than 60% or 100% additional gypsum is absolutely abundant and can decrease the strength severely when it is excessive. Because samples under a standard condition with >95% R.H., gypsum, as a material with poor water resistance, will lose its strength gradually. This property results in the outcome shows decrease of compressive strength in 7 days in the Figure 3 and Figure 4. In all groups in which quicklime was added, the compressive strength of QL20 was almost the best at all ages. So optimum content of quicklime in the double liquid grouting materials with a water/cement ratio of 1:1 is 20% [4]. The reaction between quicklime and aluminum sulfate formed the initial ettringite and it provided a supporting effect and increased the stiffness of the matrix, thus increased the compressive strength [4,43]. Higher content than 20% of quicklime decreased the compressive strength and the reason could be that the fluorgypsum was not enough, which is not good for the compressive strength of specimens. When there was not enough gypsum, the reactions in Equations (3) and (6) could be affected and there could be not enough ettringite formed at later stage after the initial ettringite formed from Equation (7).

### 3.3. Hydration Heat

**Figure 5:**
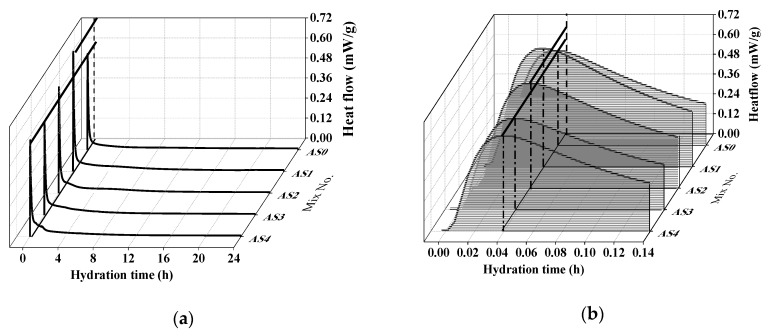
Effect of aluminum sulfate on the hydration heat flow: (**a**) 0–24 h, (**b**) 0–0.14 h.

**Figure 6:**
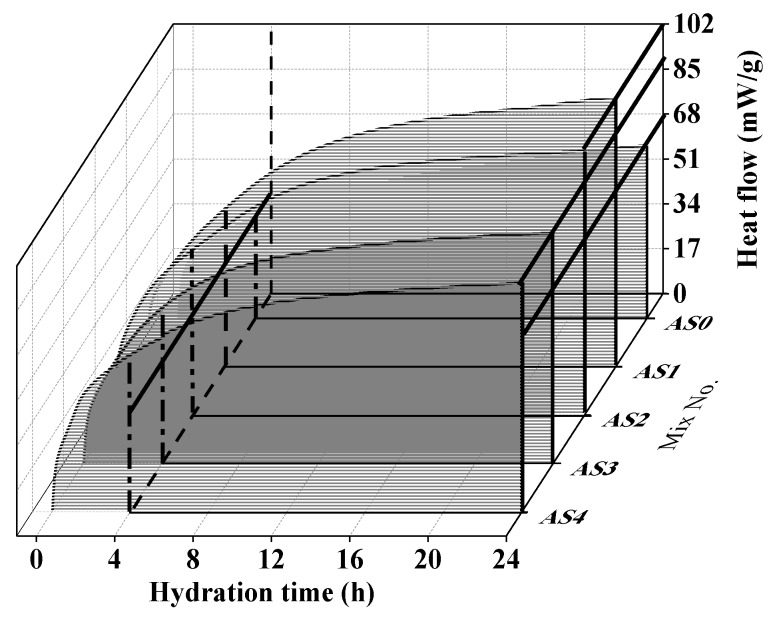
Effect of aluminum sulfate on the accumulated hydration heat.

Figure 5 shows the effect of aluminum sulfate on the hydration heat flow of the double liquid grouting materials in 24 h and the initial 8 min (0.14 h). The first peak at around 2 min was mainly because of the formation of ettringite in water. In AS1, the addition of 1% aluminum sulfate increased this peak because the reaction between aluminum sulfate, quicklime and water formed the ettringite (Equation (7)) and released more heat. In AS3 and AS4, with the increase of aluminum sulfate up to 3% and 4%, the peak decreased back to a similar position of AS0. This might suggest that with a higher content of aluminum sulfate in Slurry B, more initial AFt can generated before mixing to act as crystalline matrix, which results in a mass of new generated AFt with higher heat flow. Therefore, the rapid generation in early age influences growth of hydrates, afterwards, causing the decrease of hydration heat release. The delayed hydration heat of AS3 and AS4 can be seen at 4 h in Figure 6. The hydration heat of all mixtures with aluminum sulfate were higher than that of AS0. The total hydration heat in the 24 h was shown in Figure 6. It can be seen that all mixtures with aluminum sulfate had higher total hydration heat than that of AS0, and the highest total hydration heat was found in the mix with 1% and 2% of aluminum sulfate.

**Figure 7:**
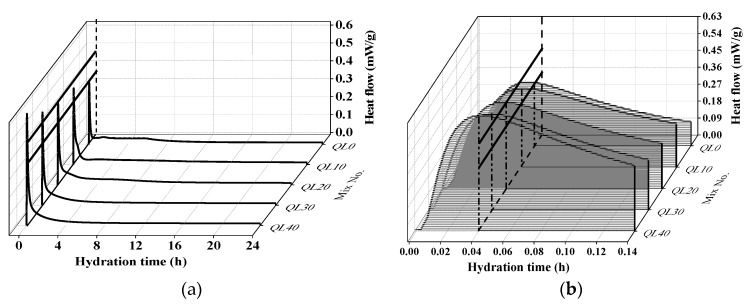
Effect of quicklime on the hydration heat flow: (**a**) 0–24 h, (**b**) 0–0.14 h.

**Figure 8:**
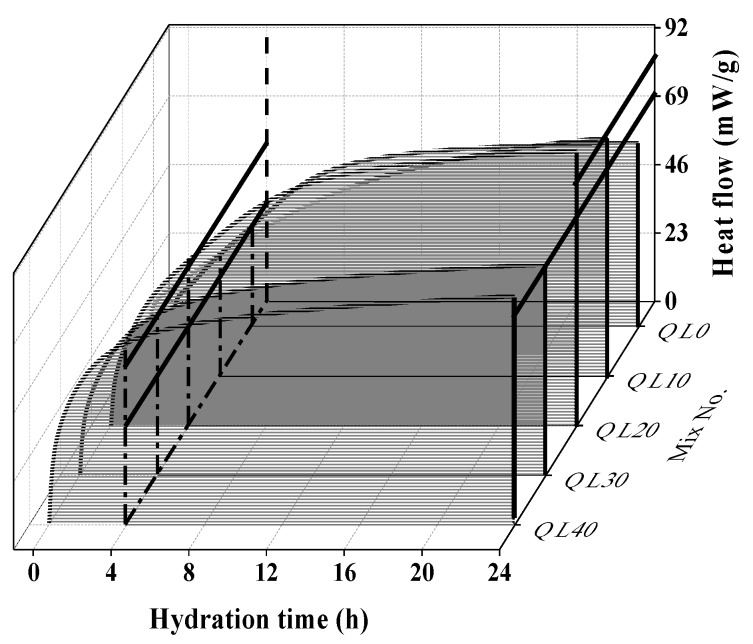
Effect of quicklime on the accumulated hydration heat.

The effect of quicklime on the hydration heat flow and total hydration heat was shown in Figure 7 and Figure 8. The first peak at around 2 min was mainly because of the dissolution of quicklime and the mix with higher content of quicklime had a higher peak. There is no quicklime in QL0, and its early hydration exothermic peak is mainly derived from the hydration of CSA cement. In Figure 8, it can be seen that QL20 had the highest total hydration heat accumulated in the 24 h, which indicated the optimum content of quicklime is 20% and this agrees with the finding reported in the compressive strength results.

### 3.4. XRD Results

**Figure 9:**
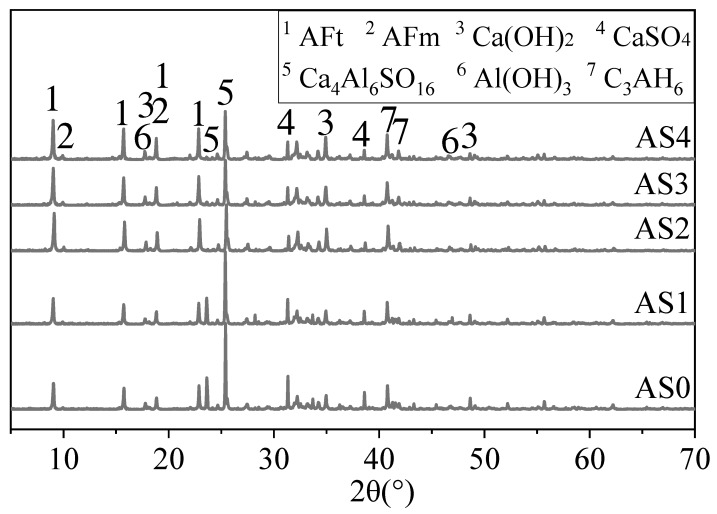
XRD patterns of the double liquid grouting materials with different aluminum sulfate contents at 1 day.

**Figure 10:**
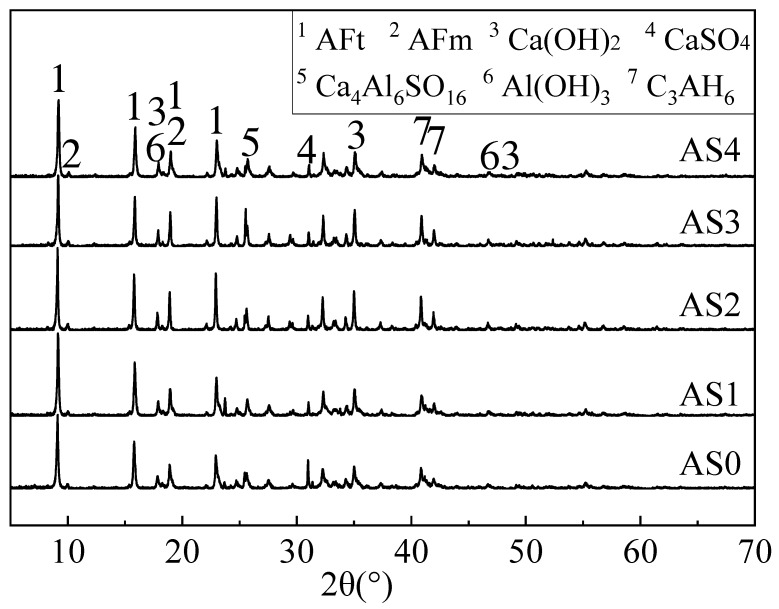
XRD patterns of the double liquid grouting materials with different aluminum sulfate contents at 28 days.

The XRD patterns of mixtures with different contents of aluminum sulfate at 1 and 28 days are shown in Figure 9 and Figure 10. Figure 9 shows that at 1 day the main hydration heat of each group of mixtures was ettringite. After adding aluminum sulfate, the characteristic peak height of gypsum decreased significantly, and the peak height ratio and peak area ratio of AFt to Ca_4_Al_6_SO_16_ at 1 and 28 days increased. This indicates that adding aluminum sulfate can promote the formation of ettringite. The peak of AFm was more obvious in AS2, AS3 and AS4. There was no significant difference in the peaks of ettringite and Ca (OH)_2_ in mixtures with 2%, 3% and 4% aluminum sulfate, and this could indicate 2% of aluminum sulfate might be the optimum content. 

Figure 10 shows that at 28 days the ettringite in all mixtures was significantly increased and the CaSO_4_ originated from fluorgypsum was significantly decreased. This indicates that more and more CaSO_4_ was reacted and additional ettringite was formed with time. In AS2, the peak ratio of ettringite to gypsum is the lowest, showing the highest hydration progress. Al(OH)_3_ was from the aluminum gel (Al_2_O_3_∙3H_2_O). By referring to the result of compressive strength in Figure 3, it suggested that both ettringite and aluminum gel contribute to a significant development of compressive strength in the double liquid grouting materials. Calcium aluminate hydrate (C_3_AH_6_ or 3CaO∙Al_2_O_3_∙6H_2_O) was observed in all mixtures and it was formed by the reaction between aluminum gel and quicklime as shown in Equation (8).

2Al(OH)_3_ + 3CaO + 3H_2_O → 3CaO∙Al_2_O_3_∙6H_2_O (8)

**Figure 11:**
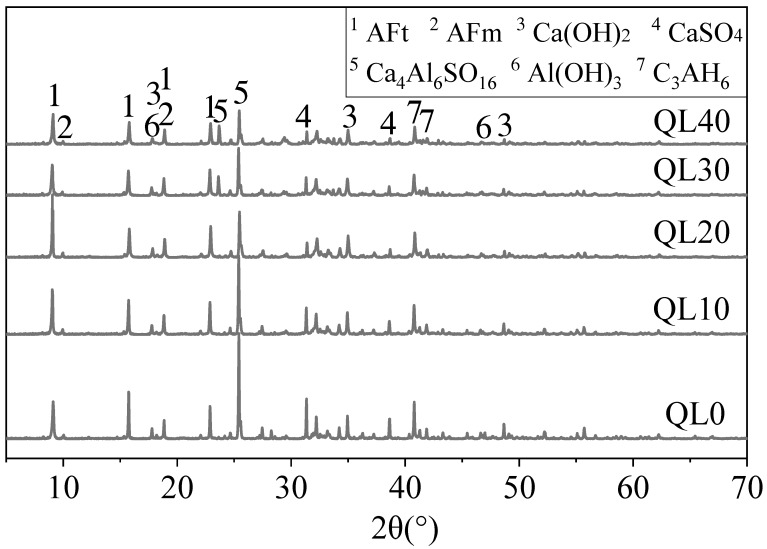
XRD patterns of the double liquid grouting materials with different quicklime content at 1 day.

**Figure 12:**
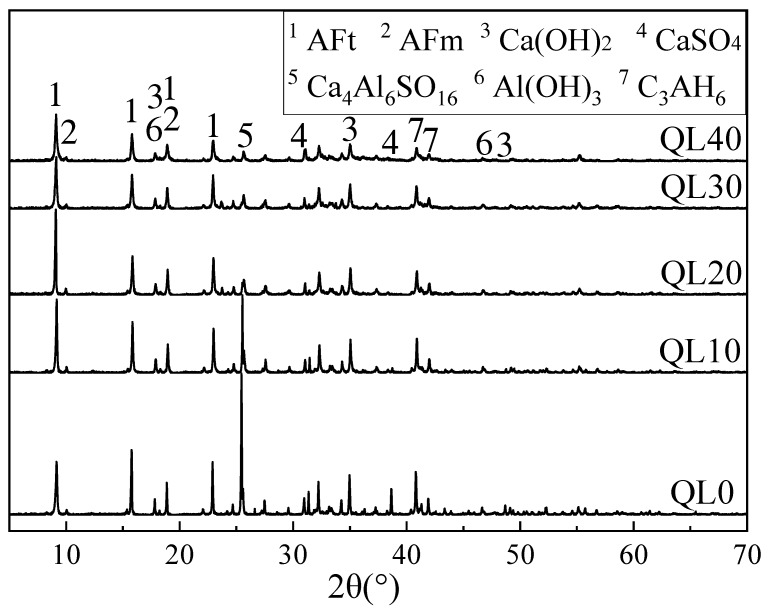
XRD patterns of the double liquid grouting materials with different quicklime contents at 28 days.

The XRD patterns of mixtures with different contents of quicklime at 1 and 28 days are shown in Figure 11 and Figure 12. In Figure 11, the characteristic peak ratio strength of ettringite and gypsum of each group was not linearly correlated with the increase of quicklime or the decrease of fluorgypsum. Among them, the peak height ratio of AFt to Ca_4_Al_6_SO_16_ in QL20 is the largest. With the increase of quicklime, the peak of Ca_4_Al_6_SO_16_ decreased and this is because the reaction between quicklime, gypsum and aluminum gel, as in Equation (6), consumed the aluminum gel and accelerated the hydration of Ca_4_Al_6_SO_16_ as in Equation (3). At the age of 28 days, both of the peak height ratio and peak area ratio of AFt to Ca_4_Al_6_SO_16_ in QL20 were the largest. Twenty percent quicklime is found to be the optimum content for the double liquid grouting materials, the reason could be that when there is not enough quicklime, the initial reaction between quicklime and aluminum sulfate (as in Equation (6)) is not sufficient and there is not enough ettringite formed initially, but when there is too much quicklime, the gypsum in slurry B is decreased and continued reaction between CSA clinker and gypsum (Equation (3)) and the reaction in Equation (6) might be affected.

### 3.5. DTA-TG Results

**Figure 13:**
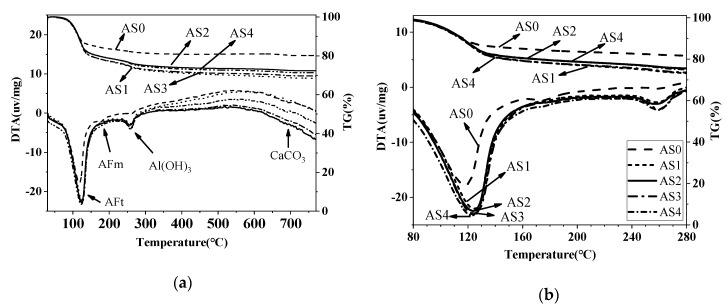
TG-DTA curves of the double liquid grouting materials with different aluminum sulfate content at 1 day: (**a**) Temperature ranges from 40–750 °C; (**b**) Temperature ranges from 80–280 °C.

**Figure 14:**
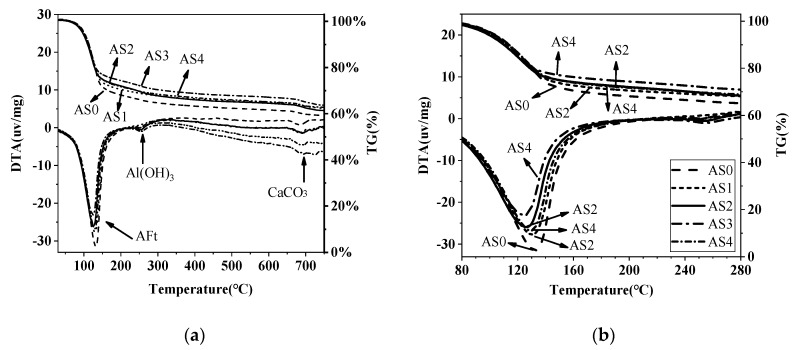
TG-DTA curves of the double liquid grouting materials with different aluminum sulfate content at 28 days: (**a**) Temperature ranges from 40–750 °C; (**b**) Temperature ranges from 80–280 °C.

Figure 13 and Figure 14 show the TG-DTA results of the double liquid grouting materials with different percentages of aluminum sulfate at the age of 1 and 28 days. The main weight loss happened at 80–150 °C, which is mainly the decomposition of ettringite (AFt) and some water loss of aluminum gel. The small peak at around 180 °C in the DTA curve is the AFm. The initial reaction between quicklime and aluminum sulfate increased the ettringite at age of 1 day compared with AS0. Overall, there was no significant difference between the weight loss of mixtures with 1–4% aluminum sulfate, and this corresponds to the similar strength of these mixtures at 1 day (Figure 13). At 250–280 °C, there was the weight losses of Al(OH)_3_. Assuming that 1 mole of ettringite is heated, 24 moles of water can be obtained in a narrow temperature range corresponding to its strong endothermic effect [44,45]. The weight losses of AFt, AFm and Al(OH)_3_ at the ages of 1 and 28 days are shown in Table 6. Figure 14 shows that, at 28 days, the weight loss of the mix with no aluminum sulfate at 80–150 °C is the highest, indicating that it has the most ettringite, aluminum gel and C-S-H, but its strength was not the highest. Instead, the mixtures in AS2 and AS3 had the greatest strength. From the XRD result (Figure 10) which shows similar content of ettringite in these mixtures, it is suggested that the highest content of ettringite and aluminum gel cannot guarantee the highest strength and the strength could also be influenced by other factors like the microstructure, which will be observed through SEM tests.

**Figure 15:**
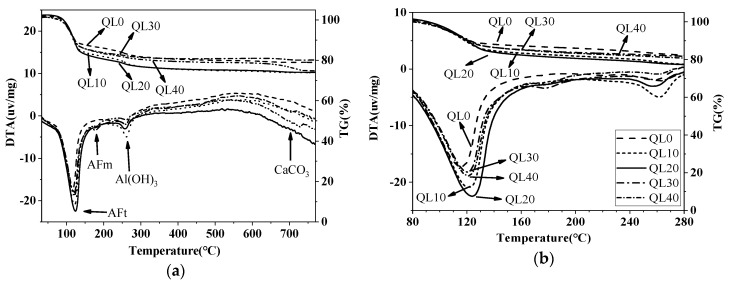
TG-DTA curves of the double liquid grouting materials with different quicklime content at 1 day: **(a**) Temperature ranges from 40–750 °C; (**b**) Temperature ranges from 80–280 °C.

**Figure 16:**
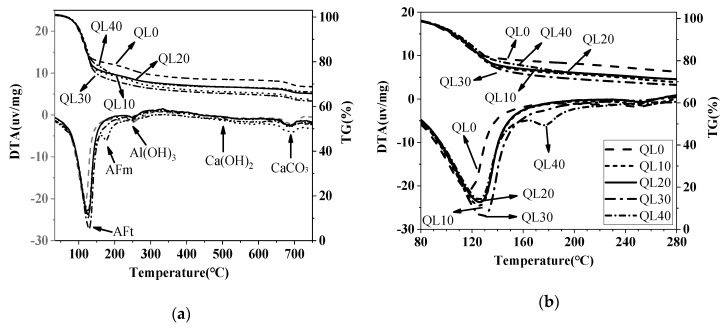
TG-DTA curves of the double liquid grouting materials with different quicklime content at 28 days: (**a**) Temperature ranges from 40–750 °C; (**b**) Temperature ranges from 80–280 °C.

Figure 15 and Figure 16 show the TG-DTA results of the double liquid grouting materials with different quicklime contents at 1 and 28 days. The weight losses of AFt, AFm and Al(OH)_3_ of the mixtures with different quicklime contents at the ages of 1 and 28 days are shown in Table 7. At 1 day, the mixture in QL20 had the highest DTA peak at 80–150 °C, which shows the highest content of ettringite formed. At 28 days, the mixtures with quicklime had higher weight loss of ettringite than that with no quicklime. The mixture in QL30 had the highest weight loss of ettringite, but its strength was lower than the mixtures in QL10 and QL30. The related mechanism will be discussed further in the discussion part.

### 3.6. SEM Results

**Figure 17:**
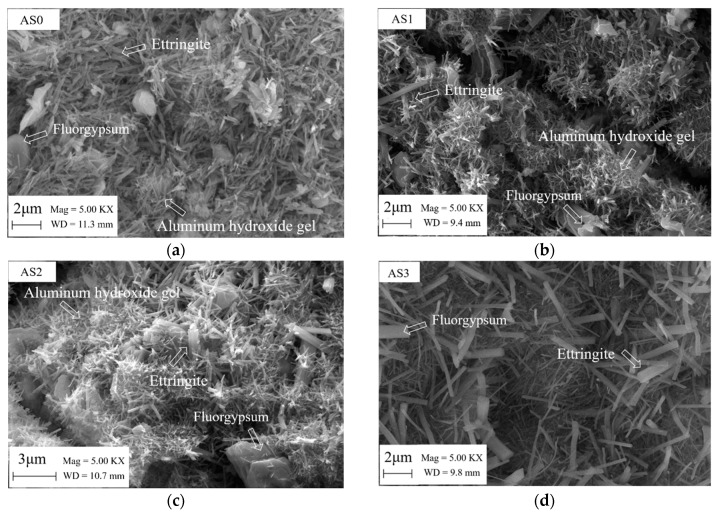

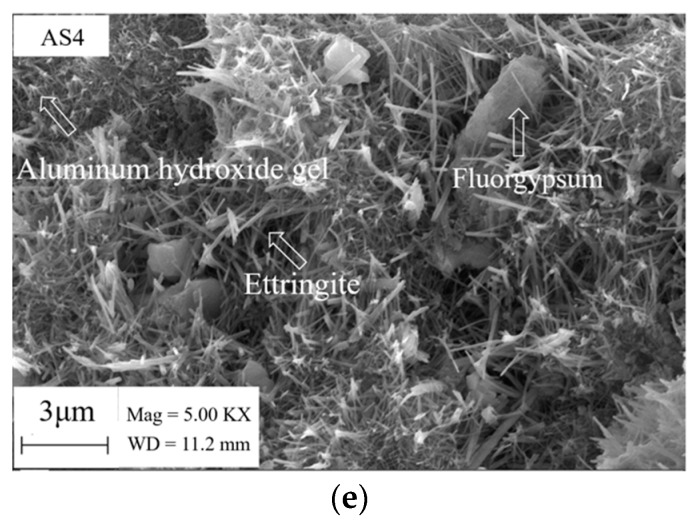
SEM images of double liquid grouting materials with different aluminum sulfate contents at 28 days: (**a**) 0%; (**b**) 1%; (**c**) 2%; (**d**) 3%; (**e**) 4%.

Figure 17 shows the SEM images of the double liquid grouting materials with different aluminum sulfate content at the age of 28 days. In mix with no aluminum sulfate, the size of ettringite formed was uniform, but in mixtures with aluminum sulfate, the sizes of ettringite are not uniform. The bigger size ettringite in mixtures with aluminum sulfate could be formed initially in slurry B before the two slurries were mixtures together, and this bigger size ettringite could contribute to a higher compressive strength [46], especially at early ages. The microstructure of mixtures in AS2 and AS3 was found to be denser than other mixtures, and that could be the reason for that the two mixtures had the highest strength at 28 days. In mixtures in AS2 and AS3, the big size ettringite acted as the main support structure, the finer size ettringite acted as the micro support structure and aluminum gel filled the pores, which gave a good resistant to external loading.

**Figure 18:**
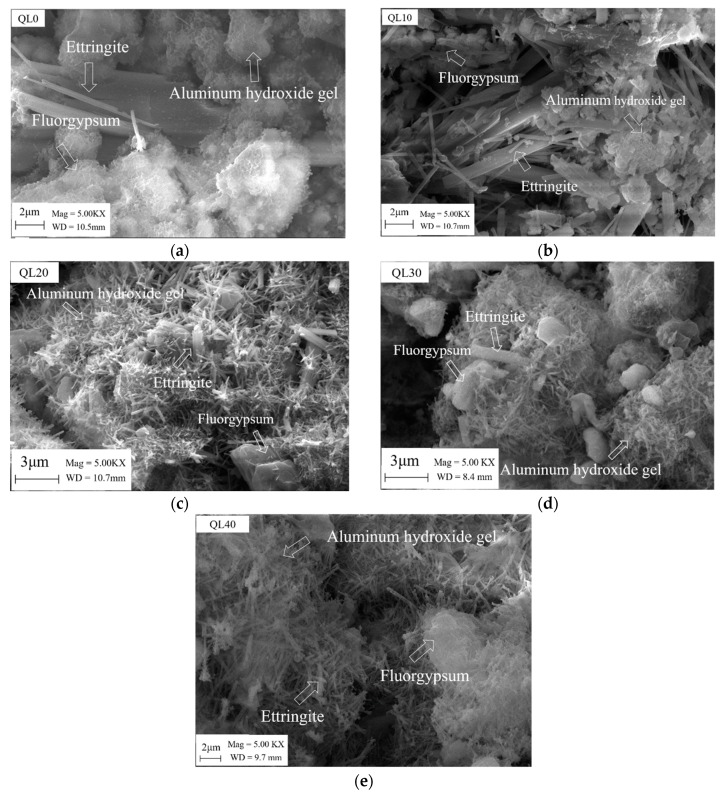
SEM images of double liquid grouting material with different quicklime contents at 28 days: (**a**) 0%; (**b**) 10%; (**c**) 20%; (**d**) 30%; (**e**) 40%.

Figure 18 shows the SEM results of the double liquid grouting materials with different quicklime contents at the age of 28 days. The mix in QL20 was found to have the most ettringite and denser structure than other mixtures. This is the reason why the mix had the highest strength. In the mixtures with not enough quicklime, there was no or not enough initially formed ettringite (bigger size), and with the hydration of CSA clinker, there was no or not enough reaction for quicklime to consume the aluminum gel and form more ettringite as in Equation (6). In mixtures with more quicklime, there might be not enough gypsum for reactions as in Equations (3) and (6). Although the initial ettringite formed in slurry B is sufficient, the continued reaction between CSA clinker and gypsum and the reaction between quicklime, aluminum gel and gypsum might be affected due to shortage of gypsum.

## 4. Discussion

### 4.1. Optimum Ratio of Quicklime/Fluorgypsum

The theoretical analysis of the optimum ratio of quicklime/fluorgypsum can be conducted through the chemical reactions between quicklime, fluorgypsum and CSA clinker (3CaO∙Al_2_O_3_∙CaSO_4_). By combing the Equation (3) and (6), Equation (9) can be obtained.

3CaO∙Al_2_O_3_∙CaSO_4_ + 8(CaSO_4_∙2H_2_O) + 6CaO + 80H_2_O → 3(3CaO∙Al_2_O_3_∙3CaSO_4_∙32 H_2_O) (9)

The optimum ratio of quicklime/fluorgypsum should be 6CaO:8CaSO_4_ by mass and it becomes (6×56):(8×136) = 23.6:76.4. This value is close to 2:8 which is obtained from our tests. The optimum content of quicklime is 23.6%, which is close to 20% as is obtained from the tests.

When the quicklime is not enough, the reaction in Equation (3) will happen and every mole of 3CaO∙Al_2_O_3_∙CaSO_4_ produces 1 mole of ettringite. When the quicklime and fluorgypsum are in the optimum ratio and the reaction in Equation (9) can happen, and every mole of 3CaO∙Al_2_O_3_∙CaSO_4_ produces 3 moles of ettringite. When the quicklime is more than enough, the fluorgypsum should be not enough and the Equation (9) can be affected and Equation (5) could happen. In Equation (5), every mole of 3CaO∙Al_2_O_3_∙CaSO_4_ produces 1 mole of ettringite. The above analysis explains the highest compressive strength in the mix with the optimum content of quicklime (20%).

### 4.2. Optimum Content of Aluminum Sulfate

The role of adding aluminum sulfate in this study is to form the initial ettringite in slurry B and thus decrease the setting time and increase the early age strength. As shown in Equation (7), the aluminum sulfate reacted with the quicklime CaO and thus could reduce the content of CaO, thereby affecting the reaction in Equation (9). Besides, from Equation (7), the ratio of CaO: ettringite is 6:1, but in Equation (9) this ratio becomes 6:3. It means it is better to have the reaction in Equation (9) than in Equation (7) in order to make most use of the quicklime. In consideration of both setting time, early strength (due to Equation (7)) and later strength as a result of the main reaction (due to Equation (9)), there is an optimum content of aluminum sulfate and this content is 2%.

## 5. Conclusions

The influence of aluminum sulfate content and the quicklime/fluorgypsum ratio on the setting time, compressive strength, hydration heat and hydration products in calcium sulfoaluminate cement-based double liquid grouting materials with a water/cement ratio of 1:1 were studied and the following conclusions can be made:

(1) Aluminum sulfate was found to not only accelerate the hydration of grouting materials but also increase the strength of grouting materials. The optimum content of aluminum sulfate in slurry B was found to be 2%. Lower content could delay the setting time, and higher content could reduce the compressive strength. The ettringite formed through reaction between aluminum sulfate and quicklime in slurry B contributed significantly to the early strength development of the double liquid grouting materials.

(2) The optimum ratio of quicklime/fluorgypsum in slurry B was found to be 2:8. Higher or lower ratio could cause insufficient of quicklime or gypsum needed for the reactions to form enough ettringite and a dense microstructure.

(3) The fluorgypsum can be used in in calcium sulfoaluminate cement-based double liquid grouting materials to replace the ordinary gypsum in order to reduce the consumption of natural gypsum.

(4) Before mixing the two slurries, ettringite was formed from the reaction between aluminum sulfate and quicklime in slurry B. After mixing the two slurries, more ettringite was formed from the reactions between CSA clinker, quicklime, and fluorgypsum. The ettringite formed acted as the supporting frames, and the aluminum gel and C-S-H acted as filling agents to support the early age strength. Fine aggregates could be considered in the mix design for providing a stable structure in the future work. Coarse aggregates are not recommended because they could decrease the fluidity and cause significant bleeding of the high water content grouting material.

## Figures and Tables

**Table 1:** Chemical composition of CSA clinker (wt.%).

SO_3_	SiO_2_	CaO	Al_2_O_3_	Fe_2_O_3_	MgO	LOI
9.44	8.59	45.21	31.68	3.86	0.82	0.4

**Table 2:** Mineral composition of CSA clinker (wt.%).

Ca_4_Al_6_SO_16_	2CaO∙SiO_2_	C_4_AF	f-SO_3_	f-CaO
64.12	25.53	6.41	1.92	2.02

**Table 3:** Chemical composition of fluorgypsum (wt.%).

SO_3_	SiO_2_	Al_2_O_3_	Fe_2_O_3_	CaO	MgO	CaF_2_	LOI
53.79	4.71	2.6	0.24	34.56	1.15	0.96	1.99

**Table 4:** Mix proportions of double liquid grouting materials.

Mix No.	Slurry A ^1^		Slurry B ^2^			
	CSA ^3^ (wt.%)	Water (wt.%)	Fluorgypsum (wt.%)	Quicklime (wt.%)	Aluminum Sulfate (wt.%)	Water (wt.%)
AS0	100	100	80	20	0	100
AS1	100	100	80	20	1	100
AS2	100	100	80	20	2	100
AS3	100	100	80	20	3	100
AS4	100	100	80	20	4	100
QL0	100	100	100	0	2	100
QL10	100	100	90	10	2	100
QL20	100	100	80	20	2	100
QL30	100	100	70	30	2	100
QL40	100	100	60	40	2	100

^1^ Slurry A is a mixture of CSA and water; ^2^ slurry B is a mixture of fluorgypsum, quicklime, aluminum sulfate and water; ^3^ CSA: CSA cement.

**Table 5:** Setting time of the slurry A with different content of aluminum sulfate.

Slurry A			Initial Setting Time (min)	Final Setting Time (min)
Aluminum Sulfate (wt.%)	CSA (wt.%)	Water (wt.%)		
0	100	100	491	619
1	100	100	489	614
2	100	100	485	607
3	100	100	486	611
4	100	100	479	599

**Table 6:** Weight loss of the double liquid grouting materials with different aluminum sulfate content.

Mix No.	1 Day Weight Loss (%)			28 Days Weight Loss (%)		
	AFt	AFm	Al(OH)_3_	AFt	AFm	Al(OH)_3_
AS0	14.45	1.82	1.76	28.88	(3.60)	2.61
AS1	18.75	(2.37) *	3.58	26.86	(3.11)	2.15
AS2	18.99	(2.34)	3.00	23.70	(2.54)	2.75
AS3	19.93	(2.85)	3.66	23.67	(2.66)	3.16
AS4	20.22	(2.46)	3.56	25.65	(3.17)	2.47

* Data in ( ) means no endothermic peak appeared.

**Table 7:** Weight loss of the double liquid grouting materials with different quicklime content.

Mix No.	1d Weight Loss (%)			28d Weight Loss (%)		
	AFt	AFm	Al(OH)_3_	AFt	AFm	Al(OH)_3_
QL0	14.24	(1.59) *	3.49	20.10	(1.90)	3.54
QL10	17.35	(2.11)	4.19	24.38	(2.56)	3.52
QL20	18.99	(2.34)	3.00	23.70	(2.54)	2.75
QL30	16.45	2.02	3.16	26.11	(3.33)	(2.49)
QL40	15.61	2.34	2.33	22.19	4.32	(2.77)

* data in ( ) means no endothermic peak appeared.
